# Medical and Philosophical Intelligence

**Published:** 1820-01

**Authors:** 


### Medical and Philosophical Intelligence.

ACCOUNT *of the Institution, in London, of an Infirmary for the Treatment of Diseases of the Skin.*—AT a meeting held at the Thatched-house Tavern, 1819, Lord JOHN RUSSELL, M.P. in the chair, it was resolved,

1st. That an Institution for the Treatment of Cutaneous Diseases is a desideratum in this kingdom, and that it is expedient one be established in a central situation of the metropolis.

2d. That the objects of the Institution be two-fold: viz. the relief of the poor labouring under cutaneous affections, and improvement in the treatment of this class of diseases.

3d. That the Institution be so regulated as to afford to medical students opportunities of acquiring a knowledge of the aforesaid diseases.

4th. That the Institution be under the direction of patrons, a president, vice-presidents, treasurer, and governors; and under the professional superintendance and management of a consulting physician, two physicians, and two surgeons.

5th. That Thomas Bateman, M.D. F.L.S. of the Royal College of Physicians of London, &c. be consulting physician.

6th. That Thomas Thomson, M.D. of the Royal College of Physicians of London, Physician Extraordinary to His Royal Highness the Duke of York, &c.; and Alexander Lyon Emerson, M.D. of the Royal College of Physicians of London, &c. be the physicians.

7th. That J. C. Carpue, Esq. F.R.S. and W. Wadd, Esq. Surgeon Extraordinary to the Prince Regent, Members of the Royal College of Surgeons of London, &c. be the surgeons.

8th. That the Institution be denominated "The Royal London and Westminster Infirmary, for the Treatment of Cutaneous Diseases."

9th. That governors at two guineas, or life subscribers of twenty guineas, shall have the privilege of sending eight patients annually; and governors at one guinea, or life subscribers of ten guineas, four patients annually.

A desire to favour the interests of so useful an Institution, has led us to insert this Report; and numerous medical men will, there can be no doubt, consider it as especially claiming their patronage: for it is the branch of pathology which is still the least understood in England, and opportunities for practical observation in it must be considered a very desiderable object. The committee have taken a house in Great Marlborough-street; though they hope the funds of the Institution will soon furnish the means of forming a larger and more convenient establishment.

It is very extraordinary, that, in London, where medical instruction is so zealously pursued, there is no chair for physiological pathology; since the method of the ordinary courses of Lectures on *Physic*, as they are termed, does not admit of an extensive application of the modern discoveries in physiology to the explanation of the phenomena of disease. Physiology alone, may be very well studied in books: it is the application of it above-mentioned that becomes so interesting and important in the chair, by means of the impressiveness here given to it, and the opportunities a good professor takes to produce a multitude of reflections and illustrative observations, which cannot be conveyed so well in any other manner. It is for these reasons that we see, in the rows at the *Val-de-Grace*, some of the most esteemed practitioners of Paris, who would not sit to hear a lecturer on physiology alone. We have been led to make these remarks by finding that Dr. Harrison has announced his intention to resume the Lectures he commenced just before his leaving England, three or four years since. They were, as we understood from an intelligent student who attended them, constituted on the principles just pointed out, and well adapted to obviate the want of which we have complained. On sitting to hear Broussais, we have had many grey heads to accompany us: we hardly know whether this may be expected in London; but it was certainly one of the most gratifying scenes we ever witnessed.

---

We some time since pointed out the "Medical Intelligencer" of Messrs. Burgess and Hill, as a publication of utility at the present time, when the number of periodical Journals embracing something relating to medicine is such, that but few persons obtain them all, and therefore lose much information of more or less importance. That pamphlet was merely of use for the purpose of reference, by giving the titles of papers; but they have recently instituted a monthly Journal,* that, though arising from the former, is of a very different character, and must prove of very considerable utility, from the specimen we have in the two Numbers which have been published. They really contain, at least hints of the nature of the contents of all the works alluded to in the title-page; and, in many instances, the substance of them is condensed in so skilful a manner, that a tolerably-perfect view of the doctrine contained in them is given; and, what is of essential importance to the utility of works of this kind, there is in it evidence of candour, liberal views, and impartiality, in the editor, that contribute much to the respectability of his talents. We say thus much, in order to direct the attention of our readers to it; and we feel confident, that those who comply with this desire, will be pleased with our recommendation.

---

* *The Medical Intelligencer, or Monthly Analytical Compendium, and Index: containing the Medical, Surgical, and Physical Contents of the Transactions of Learned Societies, of the Quarterly and Monthly Journals and Reviews, &c. forming a concentrated Record of Medical Literature, &c.* 8vo. pp. 16. Published monthly, price 6d. by Burgess and Hill, Great Windmill-street.

We had not hitherto noticed the pamphlet with which Mr. Banks favoured us some time since,* because we had waited to know the results of some enquiries respecting the application of his judicious observations to the medical police of England generally, relating to the sick poor. We have found that the evils which he has employed his useful exertions to avert, are chiefly local: we believe the attempts made a few years since to effect a general reform on this subject have been suspended; but, should this be not wholly the case, we recommend to those engaged in it the perusal of the little pamphlet of Mr. Banks, as a means of furnishing them with some useful topical information.

---

*Observations respecting the Causes of Incubus.*—Dr. Pitschaft, of Bonfeld, Hellbronn, after citing the remarks of Lommius, that *Incubus malum familiare est crepula, et multa sæpe cruditate oneratis;* and that *Vix unquam excidit in latus cubantibus;* says,† "I, in my youth, was a sleep-walker, and afterwards became afflicted with this malady (Incubus) in a very severe manner; but, by using water as drink, taking a spare supper without wine, and being careful not to lie on the right side, I have freed myself from it. This disorder, doubtless, may arise from different causes, and therefore will require different modes of treatment. I often experience an obstruction in the hepatic system, and am inclined to constipation of the bowels."

We have adduced the foregoing observations, because we have ourselves known several cases of this distressing affection to be relieved in the same manner. It should, however, be remembered, that a dilatation of the stomach with flatus will act in the same way as when it is filled with food: in some persons flatus commonly produces it. But there are too many cases which baffle every effort to relieve them: however, the mode of treating them above indicated will succeed in a very great proportion of cases; for it is from the causes above-mentioned that it very frequently arises.

---

*Account of a singular Affection of Vision,* by James Russell, Esq. F.R.S.E. and Professor of Clinical Surgery in the University of Edinburgh. Contained in a Letter to Dr. Brewster.—As I know that you take a deep interest in every curious fact connected with optics, I

---

* *Observations on the Medical Arrangements for the Relief of the Sick Poor: addressed to the Guardians, Directors, and Acting Guardians of the Poor, and to the Inhabitants of the Isle of Wight.* By W. H. Banks, Surgeon, Royal Navy. Newport, 1819; price 1s.

† In the *Journal der Practischen Heilkunde,* September 1819, amongst a collection of very interesting practical observations,—a sort of digest of his Note-book. Some physicians, as Drs. Schaeffer, Fischer, (the late, we should say, for we hear that he has lately died,) Hartmann, and others, are in the habit of furnishing Prof. Hufeland with a yearly digest of this kind;—a practice which, if more generally imitated, would furnish a multitude of useful observations that are now lost to the public, and contribute much to the improvement of practical medicine. We think it not unlikely that we may be incited to give a translation of some of the collections here alluded to, as a specimen of their manner as well as matter.

use the freedom to send you an account of a singular affection of vision, which occurred to me some years ago.

A gentleman came to town for a consultation, on account of severe complaint in his stomach. Previously to the commencement of this complaint he saw equally well with both eyes, and the focal distance of distinct vision was the same in each of them. This distance, however, had now undergone a change in both eyes; and, what is a remarkable circumstance, the change in the two eyes was in opposite directions; the distance in the one eye having become longer, and in the other shorter, than the original focal distance. But, while the two eyes no longer correspond in their limits of distinct vision, each of them still retained the power of adapting itself to variations in the distance of external objects, so far as its limits of distinct vision admitted. The pupils retained their natural contractility to the stimulus of light. The cornea, and the different humours, possessed perfect transparency, and the most careful examination did not discover the slightest appearance of disease in any part of the eye.

The gentleman referred the origin of this infection of vision to the disordered state of his stomach; and I saw no reason to entertain the smallest doubt with regard to the justness of his conclusion. The eyes readily sympathize with any morbid irritation of the stomach; though it certainly would not have been expected that one kind of irritation, proceeding from the same source, should have produced such dissimilar effects in the two eyes. The gentleman left town before the complaint in his stomach was removed, which prevented us from verifying our opinion with regard to the dependence of the affection of his eye-sight upon the state of his stomach, by following the result of the case.

The course of my reading has furnished me with only a single case at all analogous to the one above related. It is given upon the authority of Mr. Ware, who was consulted by a lady, on account of a recent discovery, that she was unable to read with the left eye. She held the book at an unusual distance; which was the more remarkable, as she had previously been short-sighted. The interposition of a convex glass of thirty-six inches focus removed all confusion. Upon taking a few medicines, to remove some constitutional disturbance, the original focus of the left eye was restored. During all the time the right eye continued short-sighted.—*Edinb. Philos. Journal*, No. 2.

### MONTHLY CATALOGUE OF MEDICAL BOOKS.

The Medical Intelligencer, or Monthly Analytical Compendium. No. II. 8d.

### REPORT OF DISEASES.

THE intense coldness of the early part of the month of December gave rise to a prevalency of pulmonic disease but rarely witnessed to such an extent at this season: the proportion of such cases has, indeed, exceeded what the ordinary rate has been in January and February for several years past in London. The most permanent forms of disease have been inflammation of the mucous membranes of the trachea and bronchiæ, in those whose lungs were previously in a healthy state; but, in persons who have shown signs of disease for some time previously, the cellular texture of the substance of the lungs has been very commonly the seat of inflammation. The latter form of disease, presenting the character of what is termed peripneumonia notha, has often been fatal in two or three days. The orthopnœa has been manifest in the most extreme degree. In some of these cases the air expelled has been found to feel cold to the hand of a person moderately warm, very soon after the attack; and these have almost universally terminated fatally. But few opportunities for dissection have occurred; but these have shown, with only a small proportion of exceptions, the existence of tubercles in the lungs, and the fatal malady has been inflammation of the cellular texture surrounding them; in some few cases extending to the pleura, and accompanied with serous effusion into the cavity of the chest. The most successful treatment has been one, and occasionally two, copious abstractions of blood from the arm at the onset of the disease, followed by leeches or cupping-glasses, and blisters, to the chest; and large doses of ipecacuanha and squills, with five grains of the mercurial pill at night; and especially the warm bath. Ipecacuanha is here more proper than antimony, because it does not so often produce purging, which should, if possible, be avoided: ipecacuanha, too, more readily and certainly acts on the mucous membranes of the lungs, producing expectoration, which is the thing to be desired. When a copious expectoration has been early produced, the disease has, in a majority of cases, terminated favourably. There is hardly any disease in which the benefits of counter-irritation are more forcibly shown, than in this very dangerous affection: when this is early produced in the mucous membranes and skin over the thorax, after the vascular system has been a little depleted, the result is often favourable; but purely depletory and antiphlogistic measures, as they are somewhat vaguely termed, will not often be successful. It is in this form of disease of the lungs that the observation of Hippocrates, and of almost every observant practitioner since him, respecting the injurious effects of purging, is so often verified: it tends to prevent the occurrence of expectoration, and thence, probably, it is that it becomes thus injurious.

Inflammation of the mucous membranes of the lungs, has been another frequent form of pulmonic disease; sometimes a coryza has appeared first, and this, in one or two days, has been followed by the catarrh. The timely exhibition of an emetic has occasionally carried off the latter affection. More free blood-letting is here necessary than in the former species of disease. In a few cases of this kind the affection of the lungs has disappeared after four, five, or six, days, and been followed by pains in the joints and about the loins: here the best practice has, apparently, been to re-excite the mucous membranes by ipecacuanha and squills, with other remedies, according to the indications of particular symptoms; and it is very interesting to observe, in many instances, how rapidly the pains arising from affection of the serous membranes have disappeared.

Those forms of disease may readily be distinguished from idiopathic pleurisy: and it is very important that this distinction should be made, for the most prompt and free blood-letting is the sheet-anchor in the latter affection.

Hooping-cough, which had been for two or three months very prevalent in the south-western district of London, has become much less common;—that is, but few recent attacks of it have been witnessed. Croup, and more general inflammation of the trachea as well as larynx, has, however, been very prevalent. Whether this has arisen from the same causes as hooping-cough, and is only a modification of the same disease, may admit of dispute; but the latter appears more like an epidemic from specific atmospheric influence, whence it is generally supposed to arise.

The other disorders have been those of the depth of winter in general; and we may conclude this report by remarking, that the City is apparently almost free from contagious maladies. A few cases of measles, scarlatina, and a very few of small-pox, are dispersed about; but London is hardly ever, perhaps never, totally exempt from the two former. Contagious fever, typhus as it is called, is extinct;—that is, there are no well-determined instances of its origin from contagion at the present time; and it is not of frequent occurrence.

### METEOROLOGICAL JOURNAL.

**By Messrs. William Harris and Co. 50, Holborn, London.**

***From November 20 to December 19, inclusive.***

| Day of Month. | Moon. | Rain gauge | THERM. | | | BAROM. | | De Luc's HYGROM Dry | Damp | | WIND. | | ATMOSPHERIC VARIATION. | | |
|---|---|---|---|---|---|---|---|---|---|---|---|---|---|---|---|
| Nov | | | | | | | | | | | | | | | |
| 20 | | | 35 | 40 | 37 | 29·82 | 29·75 | | 4 | 5 | WNW | W | Cloud. | Cloud. | Rain |
| 21 | | ,09 | 37 | 40 | 38 | 29·75 | 29·32 | | 4 | 6 | WNW | SW | Cloud. | Cloud. | Sho'ry |
| 22 | | ,03 | 40 | 43 | 33 | 29·21 | 29·43 | | 8 | 7 | WSW | N | Foggy | Cloud. | Fine |
| 23 | | | 35 | 38 | 31 | 29·54 | 29·67 | | 6 | 5 | NW | W | Cloud. | Fine | Fine |
| 24 | ☾ | | 33 | 36 | 29 | 29·80 | 29·84 | | 6 | 5 | NW | NW | Cloud. | Fine | Foggy |
| 25 | | | 31 | 37 | 33 | 29·93 | 30·04 | | 6 | 8 | NW | NNW | Fine | Cloud. | Fine |
| 26 | | | 34 | 39 | 36 | 29·82 | 29·90 | | 7 | 8 | WNW | SW | Foggy | Fine | Foggy |
| 27 | | | 38 | 40 | 32 | 29·95 | 29·92 | | 9 | 9 | NW | WNW | Foggy | Cloud. | Fine |
| 28 | | ,08 | 34 | 35 | 42 | 29·87 | 29·70 | | 8 | 10 | W | SSW | Foggy | Foggy | Rain |
| 29 | | ,19 | 49 | 50 | 50 | 29·54 | 29·73 | | 12 | 11 | WSW | WSW | Rain | Rain | Cloud. |
| 30 | | ,10 | 52 | 53 | 48 | 29·65 | 29·59 | | 12 | 12 | S | S | Cloud. | Cloud. | Rain |
| Dec | | | | | | | | | | | | | | | |
| 1 | ○ | ,20 | 49 | 49 | 42 | 29·86 | 30·10 | | 12 | 15 | NW | SSW | Rain | Cloud. | Fine |
| 2 | | ,25 | 49 | 49 | 37 | 29·97 | 30·10 | | 17 | 13 | SW | WNW | Rain | Rain | Fine |
| 3 | | | 39 | 40 | 38 | 30·20 | 30·20 | | 10 | 9 | WNW | WSW | Fine | | |
| 4 | | ,40 | 42 | 44 | 40 | 29·79 | 29·63 | | 8 | 11 | NW | NE | Rain | Rain | Cloud. |
| 5 | | | 41 | 42 | 37 | 29·90 | 30·00 | | 10 | 10 | NE | ENE | Foggy | Cloud. | Fine |
| 6 | | | 38 | 39 | 33 | 30·08 | 30·10 | | 9 | 9 | ENE | E | Fine | Cloud. | Sleet |
| 7 | | ,05 | 36 | 37 | 30 | 30·03 | 30·00 | | 10 | 8 | E | E | Rain | Sleet | Cloud. |
| 8 | | | 31 | 31 | 25 | 30·05 | 30.10 | | 6 | 5 | E | ENE | Fine | | |
| 9 | ☽ | ,05 | 27 | 28 | 29 | 30·10 | 30·02 | | 5 | 7 | E | ESE | Fine | Fine | Snow |
| 10 | | | 30 | 31 | 22 | 29·94 | 30·03 | | 8 | 7 | N | NW | Cloud. | Fine | Fine |
| 11 | | | 23 | 26 | 26 | 30·03 | 30·06 | | 7 | 9 | WNW | W | Cloud. | Fine | Cloud. |
| 12 | | | 30 | 35 | 29 | 29·93 | 29·87 | | 10 | 12 | WSW | WNW | Fine | Fine | Cloud. |
| 13 | | | 30 | 35 | 27 | 29·80 | 29·80 | | 11 | 11 | W | WNW | Fine | | |
| 14 | | | 30 | 34 | 31 | 29·70 | 29·55 | | 10 | 8 | WNW | WNW | Cloud. | Fine | Fine |
| 15 | | | 33 | 34 | 33 | 29·50 | 29·63 | | 11 | 13 | W | WNW | Fine | Cloud. | Fine |
| 16 | | | 35 | 37 | 36 | 29·93 | 30·02 | | 13 | 14 | W | SSW | Fine | Fine | Cloud. |
| 17 | ● | ,33 | 40 | 41 | 19 | 29·70 | 29·43 | | 17 | 27 | S | WSW | Rain | Rain | Cloud. |
| 18 | | | 51 | 53 | 51 | 29·43 | 29·76 | | 54 | 31 | W | W | Fine | Fine | Cloud. |
| 19 | | | 53 | 57 | 53 | 29·81 | 29·78 | | 30 | 28 | SW | WSW | Cloud. | Fine | Cloud. |

The quantity of rain fallen in November, is 1 inch and 80-100ths.

*** *The publication of the* Supplementary Number *to the present Volume of the* London Medical and Physical Journal, *is deferred until the latter end of January, in order that it may comprise a complete View of English Medical Literature to the termination of the Year* 1819, *in England, and of that of Foreign Nations as nearly as possible to the same period. It will be delivered with the February Number of the Journal to those who order it from their respective Booksellers.*

*The* General Index to this Journal *was published on the 20th of December, and is ready for delivery.*

